# SETDB2 promoted breast cancer stem cell maintenance by interaction with and stabilization of ΔNp63α protein

**DOI:** 10.7150/ijbs.43611

**Published:** 2020-05-18

**Authors:** Liu Ying, Xie Fei, Li Jialun, Xiao Jianpeng, Wang Jie, Mei Zhaolin, Fan Hongjia, Fang Huan, Li Sha, Wu Qiuju, Yuan Lin, Liu Cuicui, Peng You, Zhao Weiwei, Wang Lulu, Wong Jiemin, Li Jing, Feng Jing

**Affiliations:** 1 Anhui University of Science and Technology Affiliated Fengxian Hospital, Shanghai 201499, China; 2 Department of Laboratory Medicine & Central Laboratory, Southern Medical University Affiliated Fengxian Hospital, Shanghai 201499, China; 3 The Third School of Clinical Medicine, Southern Medical University, Guangdong Province, Guangzhou 510515, China; 4 Shanghai University of Medicine & Health Sciences, Affiliated Sixth People's Hospital South Campus, Shanghai 201499, China; 5 Joint Research Center for Precision Medicine, Shanghai Jiao Tong University & Affiliated Sixth People's Hospital South Campus, Shanghai 201499, China; 6 Department of clinical laboratory, Taihe Hospital, Hubei University of Medicine, 29 South Renmin Road, Shiyan, Hubei 442000, China; 7 Shanghai Key Laboratory of Regulatory Biology, Institute of Biomedical Sciences and School of Life Sciences, East China Normal University, Shanghai 200241, China

**Keywords:** Breast cancer stem cell, SETDB2, Hedgehog pathway, ΔNp63α, stability

## Abstract

The histone H3K9 methyltransferase SETDB2 is involved in cell cycle dysregulation in acute leukemia and has oncogenic roles in gastric cancer. In our study, we found that SETDB2 plays essential roles in breast cancer stem cell maintenance. Depleted SETDB2 significantly decreased the breast cancer stem cell population and mammosphere formation *in vitro* and also inhibited breast tumor initiation and growth *in vivo*. Restoring SETDB2 expression rescued the defect in breast cancer stem cell maintenance. A mechanistic analysis showed that SETDB2 upregulated the transcription of the ΔNp63α downstream Hedgehog pathway gene. SETDB2 also interacted with and methylated ΔNp63α, and stabilized ΔNp63α protein. Restoring ΔNp63α expression rescued the breast cancer stem cell maintenance defect which mediated by SETDB2 knockdown. In conclusion, our study reveals a novel function of SETDB2 in cancer stem cell maintenance in breast cancer.

## Introduction

Breast cancer stem cells (BCSCs), as a subpopulation of cancer cells with stem cell properties, play crucial roles in tumorigenesis, metastasis, relapse and therapy resistance 1-4. These cancer stem cells (CSCs), like normal stem cells, retain self-renewal capacity and can differentiate into various tumor cell populations 5, 6. Their highly aggressive characteristics and the lack of effective therapeutic strategies make BCSCs a high priority in the investigation of the molecular mechanisms governing cancer stem cell properties.

TP63 is a member of the P53 family and expresses as multiple protein isoforms 7. The use of alternative transcription start sites (TSSs) produces transactivating isoforms (TAp63) with an N-terminal transactivation domain (TAD) and ΔN isoforms (ΔNp63) without TAD. Both the TAp63 and ΔNp63 transcripts have alternative C-terminal splicing (α, β, and γ) 7. ΔNp63α is the most abundant isoform which expressed in the majority of epithelial tissues including the mammary epithelium 8. In cancer research, ΔNp63α has been identified as an oncogene which is indispensable for cancer stem cell growth and tumorigenesis 9. In human breast cancer stem cell populations, ΔNp63α expression is highly enriched 10. In mouse model of basal-type breast cancer tumorigenesis, ΔNp63 can govern the tumor-initiation activity of breast cancer cells by directly driving FZD7 expression to enhance WNT signaling pathway 11. Downregulation of ΔNp63 in MMTV-ErbB2-derived mammospheres significantly inhibits the self-renewal capacity of breast cancer stem cells and tumor growth 12. In terms of its molecular mechanism, ΔNp63 can directly control the transcription of Sonic Hedgehog (SHH), GLI family zinc finger 2 (GLI2) and Patched1 (PTCH1) to enhance the stemness-associated pathway-- Hedgehog signaling pathway 12.

H3K9 methylation is a common histone marker associated with transcriptional repression. SETDB2 (CLLD8 or KMT1F) is a member of the histone H3K9 methyltransferase family-KMT1 sub-family, which includes SUV39H1, SUV39H2, G9a and SETDB1 13-15. SETDB2 plays important roles in immune system 15-17 and embryonic development 18, 19. In cancer research, SETDB2 has been found to be involved in cell cycle dysregulation in acute leukemia 20, associated with the prognosis and metastasis of renal tumors 21, and plays an oncogenic role in gastric cancer 22. However, the roles and mechanism of SETDB2 in cancer stem cells and breast cancer are not clear.

In our study, we found that the SETDB2 expression level was significantly increased in mammospheres of breast cancer cells. SETDB2-knockdown significantly decreased the percentage of ALDH^+^ cells and mammosphere formation *in vitro*. In a mouse model, silencing of SETDB2 also decreased breast cancer initiation and tumor growth. Restoring SETDB2 expression reversed the loss of breast cancer stem cell maintenance observed upon SETDB2-knockdown. The mechanistic analysis suggested that SETDB2 upregulated the transcription of the ΔNp63α downstream Hedgehog pathway genes. SETDB2 also interacted with and methylated ΔNp63α, stabilized ΔNp63α protein. Restoring ΔNp63α expression in SETDB2-knockdown breast cancer cells rescued the breast cancer stem cell maintenance. Thus, our study reveals a novel role of SETDB2 in breast cancer stem cell maintenance.

## Materials and Methods

### Plasmids, primers and antibodies

The shRNA lentivirus plasmid negative control (NC), SETDB2-knockdown constructs, lentivirus plasmid Plvx-IRES-Neo-Flag-SETDB2, and the eukaryotic expression plasmid pcDNA3.1-HA-ΔNp63α was purchased from TranSheepBio (Shanghai, China). The eukaryotic expression plasmid Myc-Ub, Myc-WWP1, and Flag-ΔNp63α was provide by Prof. Ceshi Chen from the Kunming Institute of Zoology of CAS. The eukaryotic expression plasmid pcDNA3.1-HA-SETDB2 was constructed using lentivirus plasmid Plvx-IRES-Neo-Flag-SETDB2 as PCR template. The sequence of SETDB2 shRNA shS-3 is 5'- CCAGGAACACAATTAGGATAT-3'. The sequence of SETDB2 shRNA shS-4 is 5'- GTTTGAAGATAATCTGCTGAT-3'. The hSETDB2 sequence is shown in **Supplementary Table 1.** The primer sequences used for Q-PCR and RT-PCR are shown in **Supplementary Table 2.**

Antibodies against SETDB2 (#A7391, Abclonal Technology, China), ΔNp63 (#6782S, Cell Signaling Technology, USA), GAPDH (#ab9485, Abcam, UK), Flag (#AF0036, Beyotime, China), HA (#51064-2-AP, Proteintech, China), Myc (#AE010, Abclonal Technology, China), H3-pan (#CS204377, Millipore, USA), H3K9me1 (#ab9045, Abcam, UK), H3K9me2 (#ab1220, Abcam, UK) and H3K9me3 (#pAb-056-050, Diagenode, Belgium) were used for Western blot. Antibodies against SETDB2 (#PA5-30848, Invitrogen, USA) and HA (#51064-2-AP, Proteintech, China) were used for Immunoprecipitation.

### Generation of stable cell lines

SETDB2-knockdown, SETDB2-rescued and ΔNp63α-rescued stable SUM159PT or MDA-MB-231 cells lines were established by lentivirus infection 23. Positive cells were screened using puromycin (#A1113803, Invitrogen, USA) or G418 Sulfate (#11811031, Invitrogen, USA) for two weeks. SETDB2-knockdown efficiency, SETDB2 and ΔNp63α overexpression level were analyzed by Q-PCR and Western blot analysis.

### RNA extraction and PCR

Total RNA was extracted using TRIzol reagent (15596018, Ambion, USA). Total RNA was then reverse transcribed to cDNA by a PrimeScript^TM^ RT reagent kit (#RR047A, TaKaRa, Japan). Q-PCR was performed using Power SYBR Green PCR Master Mix (#4367659, Applied Biosystems, USA). RT-PCR was performed using Taq DNA Polymerase (#EP0402, Thermo Fisher, USA).

### Western blot

Cells were collected by centrifugation and were then lysed in RIPA buffer. Then, the proteins were separated by SDS-PAGE after which the proteins were transferred to Bio Trace^TM^ NT nitrocellulose membranes (#25312915, Pall Corporation, USA). The membranes were incubated with primary and secondary antibodies, and the signals were visualized with a Tanon^TM^ Highly-sig ECL western blotting substrate Reagent kit (#180-5001, Tanon, China).

### Immunoprecipitation

For immunoprecipitation, cells were collected by centrifugation and were lysed in lysis buffer (50 mM Tris-HCl, pH 7.4, 150 mM NaCl, 1 mM EDTA, 1% Triton X-100, protease inhibitor cocktail (#P8340, Sigma-Aldrich, Germany)) on ice for 30 mins. Then, the cell lysate supernatant was incubated with antibodies and protein A-agarose beads by rotation overnight at 4°C. For Flag-tag immunoprecipitation, the cell lysate supernatant was incubated with anti-FLAGs M2-agarose beads (#A2220, Sigma-Aldrich, Germany). After washing, the proteins were resuspended in SDS loading buffer and analyzed by Western blot.

### Protein stability analysis

The cells were treated with 50μg/mL protein synthesis inhibitor Cycloheximide (CHX) for 0, 4, 8, and 12 hours, or the cells were treated with 20 μM proteasome inhibitor MG132 for 12 hours. Finally, the cells were collected and analyzed by Western blot.

### Protein ubiquitination assay

293T cell were co-transfected with expressing plasmids for Myc-Ub, Myc-WWP1, Flag-ΔNp63α and HA-SETDB2 in 6-well plates. 48h after transfection, the cells were treated with 20 μM proteasome inhibitor MG132 for 8 hours to accumulate the ubiquitinated ΔNp63α. Then the cell were harvested in buffer A(150mmol/L NaCl, 0.1% NP-40, 50mmol/L Tris/HCl pH7.9, 5mmol/L EDTA, 10% glycerol, 0.5% SDS and protease inhibitor cocktail (#P8340, Sigma-Aldrich, Germany)). The samples were boiled for 20mins. 100μL sample were diluted with 400μl buffer B(150mmol/L NaCl, 0.1% NP-40, 50mmol/L Tris/HCl pH7.9, 5mmol/L EDTA, 10% glycerol and protease inhibitor cocktail (#P8340, Sigma-Aldrich, Germany)) and incubated with 10μL anti-FLAGs M2-agarose beads (#A2220, Sigma-Aldrich, Germany) overnight at 4°C with rotation. After washing, the proteins were resuspended in SDS loading buffer and analyzed by Western blot. The ubiquitin modified ΔNp63α proteins and WWP1 were detected by anti-Myc antibody. Flag-ΔNp63α was detected by anti-Flag antibody. HA-SETDB2 was detected by anti-HA antibody. Anti-GAPDH Antibody used as loading control.

### In vivo methylation assay

Flag-ΔNP63α was co-transfected with HA-SETDB2 or Vector as control. The cells were collected 48 hr after transfection and lysed in denaturing lysis buffer (50 mM Tris-HCl pH 8.0, 1 mM EDTA, 1% SDS and protease inhibitor cocktail (#P8340, Sigma-Aldrich, Germany)). The lysates were heated at 95°C for 20 mins and clarified by centrifugation at 12,000 rpm for 20 mins at room temperature. The supernatant was diluted five times with non-denaturing buffer (50 mM Tris-Cl, pH8.0, 150mM NaCl, 1% NP-40, 1mM EDTA and protease inhibitor cocktail (#P8340, Sigma-Aldrich, Germany)), and the lysates were incubated with anti-FLAGs M2-agarose beads (#A2220, Sigma-Aldrich, Germany) for 3 hr at 4°C. After extensive washing with non-denaturing buffer, complexes were boiled in SDS loading buffer and analyzed by Western blot.

### Mammosphere assay

The NC, SETDB2-knockdown, SETDB2-rescued and ΔNp63α-rescued SUM159PT or MDA-MB-231 cells were subjected to a primary mammosphere assay according to a previously described protocol 24. For the secondary mammosphere assay, primary mammospheres derived from SUM159PT cells were collected and digested in 2 mg/mL DNaseI (#10104159, Sigma-Aldrich, Germany) and 4 mg/mL collagenase type IV (#5138, Sigma-Aldrich, Germany), after which the cells were counted and seeded in ultra-low attachment plates according to a previously described protocol 24.

### ALDEFLUOR™ Assay

The NC, SETDB2-knockdown and SETDB2-rescued SUM159PT and MDA-MB-231 cells were first trypsinized. Then, 1 × 10^6^ live cells were counted using a Trypan blue assay. The ALDEFLUOR™ assay was performed using an ALDEFLUOR^TM^ Kit (#01700, StemCell Technologies, Canada) according to the manufacturer's instructions. Samples were then analyzed by flow cytometry (BD Biosciences, Franklin Lakes, USA).

### Cell proliferation and colony formation assays

The NC and SETDB2-knockdown SUM159PT and MDA-MB-231 cells were seeded and used for CCK-8 and colony formation assays according to a previously described protocol 24.

### Orthotopic mammary adenocarcinoma xenografts

Using a protocol approved by the Ethics Committee of East China Normal University, NC or SETDB2-knockdown SUM159PT cells were injected into the right mammary fat pads of nude mice (1×10^6^ cells per gland). The tumor sizes were monitored every week with a caliper and were calculated as tumor volume=Length× Width^2^×0.52. All mice were sacrificed at the end of the experiment, and the tumors were collected for analysis. For the extreme limiting dilution analysis (ELDA), 60 mice were separated randomly into 12 groups (five mice per group). The right fourth mammary fat pads of the nude mice were injected with NC and SETDB2-knockdown SUM159PT cells at concentrations of 5×10^5^, 5×10^4^, 5×10^3^ and 5 ×10^2^ cells per gland. All the mice were sacrificed at the end of the experiment, and the tumors were collected for analysis as previously described 25.

### Statistical analysis

SPSS version 17.0 software was used for the statistical analysis. The results were expressed as the mean ± standard deviation (SD) or as the mean ± standard error of the mean (SEM). Student's t-test was used to estimate the significant differences between groups. In all experiments, *P* < 0.05 was considered statistically significant.

## Results

### SETDB2-knockdown reduces cancer stem cells in breast cancer

**Figure 1:**
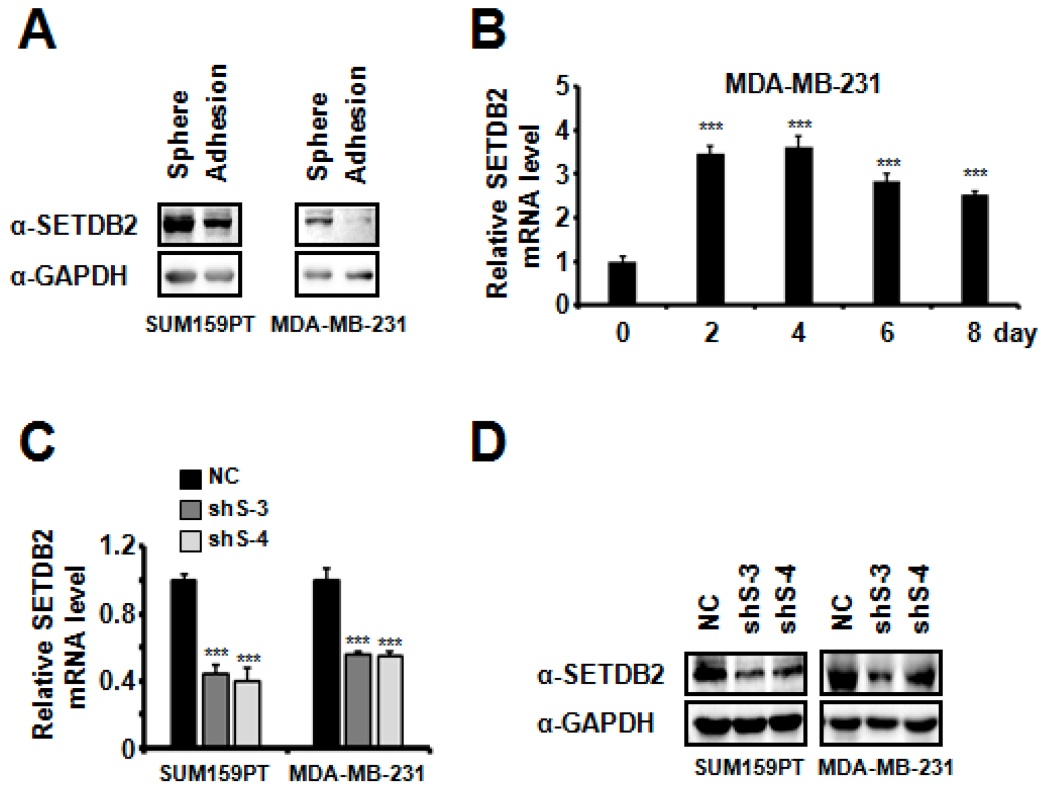
** SETDB2 expression analysis in adherent cells and mammospheres derived from breast cancer cells.** (**A**) SETDB2 protein levels in SUM159PT and MDA-MB-231 cells cultured as adherent monolayers or as mammospheres were measured by Western blot. (**B**) MDA-MB-231 cells were cultured as mammospheres for 0, 2, 4, 6 and 8 days. The SETDB2 mRNA level was measured by Q-PCR. Q-PCR (**C**) and Western blot (**D**) analysis of SETDB2-knockdown efficiency in SUM159PT and MDA-MB-231 stable cell lines. (NC: Negative Control; shS-3: shSETDB2-3; shS-4: shSETDB2-4; Data are presented as the mean ±SD of three independent experiments. ***P < 0.005)

In order to explore the epigenetic regulation of breast cancer stem cells (BCSCs), we examined the transcription level of 25 histone methyltransferases in MDA-MB-231 cells grown under adherent or sphere-forming conditions (non-adherent conditions in defined serum-free media) 26-28. We found that SETDB2 was significantly increased in mammospheres (data not shown). Then, we further examined the protein level of SETDB2 in adherent cells and spheres of SUM159PT and MDA-MB-231 cells. The SETDB2 protein level was significantly increased in the sphere group (**Figure 1A**). We also observed a similar elevation of the SETDB2 transcription level when MDA-MB-231 cells were induced to form mammospheres under sphere-forming conditions (**Figure 1B**). These data suggest that SETDB2 may exert a positive effect in BCSC maintenance.

**Figure 2:**
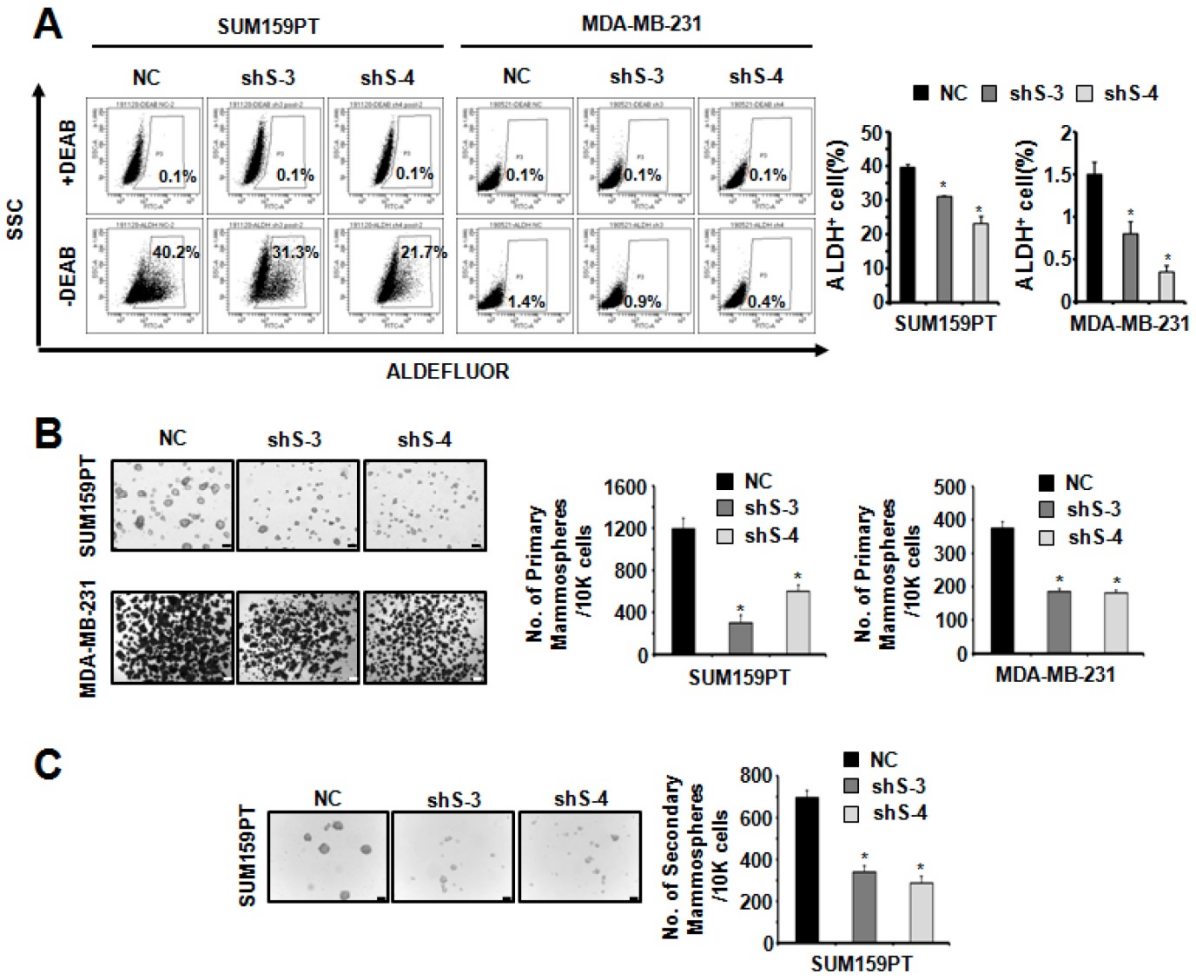
** SETDB2-knockdown reduced the percentage of cancer stem cells.** (**A**) Representative results of FACS analysis of ALDH activity for SETDB2-knockdown SUM159PT and MDA-MB-231 cell lines. The percentage of ALDH^+^ cells was quantified and is shown on the right. (**B**) Representative microphotographs (bar, 100 μm and 200 μm) of primary mammospheres derived from SUM159PT and MDA-MB-231 SETDB2-knockdown cells. Mammospheres with a diameter greater than 50 μm were counted and quantified, as shown on the right. (**C**) Representative microphotographs (bar, 100 μm) of secondary mammospheres derived from SUM159PT SETDB2-knockdown cells. Mammospheres with a diameter greater than 50 μm were counted and quantified, as shown on the right. (NC: Negative Control; shS-3: shSETDB2-3; shS-4: shSETDB2-4; Data are presented as the mean ±SD of three independent experiments. *P < 0.05)

To study the function of SETDB2 in BCSCs, we examined the protein levels of SETDB2 in several breast cancer cell lines and found that SETDB2 was expressed in most breast cancer cell lines (**Figure S1A**). Considering the self-renewal and tumor formation ability of breast cancer cell lines, we chose SUM159PT and MDA-MB-231 to generate stable SETDB2-knockdown cell lines. The knockdown efficiency was analyzed by Q-PCR (**Figure 1C**) and Western blot (**Figure 1D**). Interestingly, the percentage of ALDH^+^ cells was significantly decreased in SUM159PT and MDA-MB-231 SETDB2-knockdown cells compared with negative control (NC) cells (**Figure 2A**). In sphere-forming conditions, the number and size of primary mammospheres derived from the SETDB2-knockdown group were also smaller than in the NC group (**Figure 2B**). The data on the secondary mammosphere formation assay in SUM159PT cells were also consistent with primary mammosphere formation (**Figure 2C**). In addition, we examined proliferation of SETDB2-knockdown SUM159PT and MDA-MB-231 cells by colony formation (**Figure S2A**) and CCK8 assays (**Figure S2B**). SETDB2-knockdown showed less of an effect on the proliferation of breast cancer cells. All the above results suggest that SETDB2 is correlated with breast cancer stem cell maintenance.

### SETDB2-knockdown inhibits tumorigenesis and tumor growth in an orthotopic model of breast cancer

**Figure 3:**
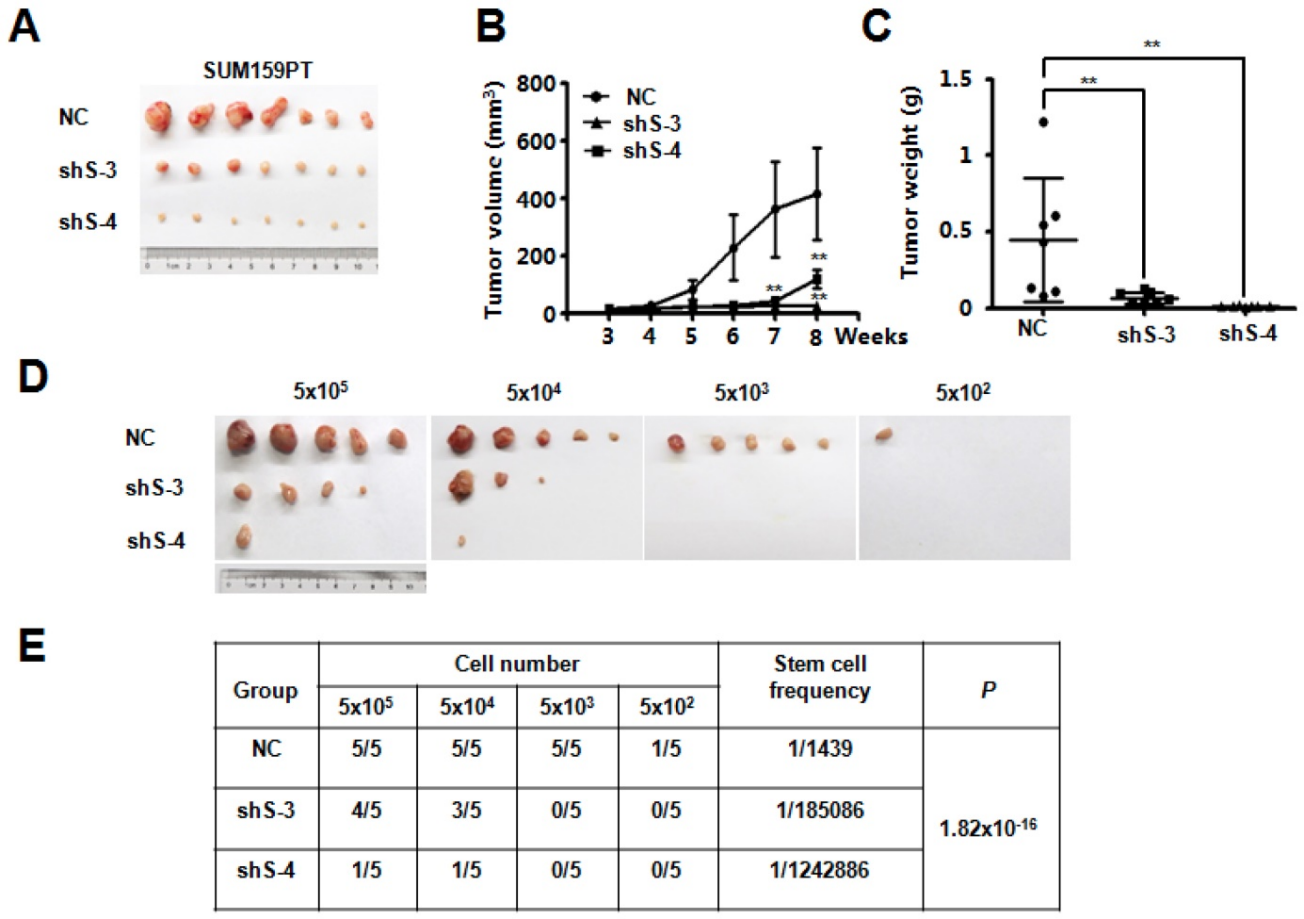
** SETDB2-knockdown decreased breast cancer tumorigenesis and tumor growth in vivo.** Nude mice were injected orthotopically with NC or SETDB2-knockdown SUM159PT cells. The tumor size is shown in (**A**). Tumor growth was monitored and statistically analyzed in (**B**) (n=7, mean ± SEM, **P < 0.01). Tumor weights were measured and statistically analyzed in (**C**) (n=7, mean ± SD, **P < 0.01). (**D**) Tumor formation in nude mice inoculated orthotopically with diminishing numbers of NC or SETDB2- knockdown SUM159PT cells (n=5). (**E**) The stem cell frequency and *P* value were determined by the ELDA.

To explore the potential function of SETDB2 in breast cancer, we examined SETDB2 function in tumor growth *in vivo*. NC or SETDB2-knockdown SUM159PT cells were inoculated into the mammary glands of nude mice, and tumor growth was monitored for up to 9 weeks. Compared with the NC group, the SETDB2-knockdown group showed a significant delay in tumor growth as well as smaller tumor size, volume and weight (**Figure 3A**, **3B** and** 3C**). To further determine whether SETDB2 silencing decreases the number of tumor initiating cells (cancer stem cells), we performed an extreme limiting dilution analysis (ELDA) 25 and found that silencing SETDB2 decreased the breast cancer stem cell frequency from 1 in 1,439 to 1 in 185,086 (sh S-3) or 1 in 1,242,886 (sh S-4), which represents a 128-fold (sh S-3) and a 863-fold (sh S-4) decrease (p=1.82×10^-16^) (**Figure 3D and 3E**). Therefore, SETDB2 plays an essential role in breast tumor initiation and growth *in vivo*.

### Restoring the SETDB2 level rescues BCSC maintenance

**Figure 4:**
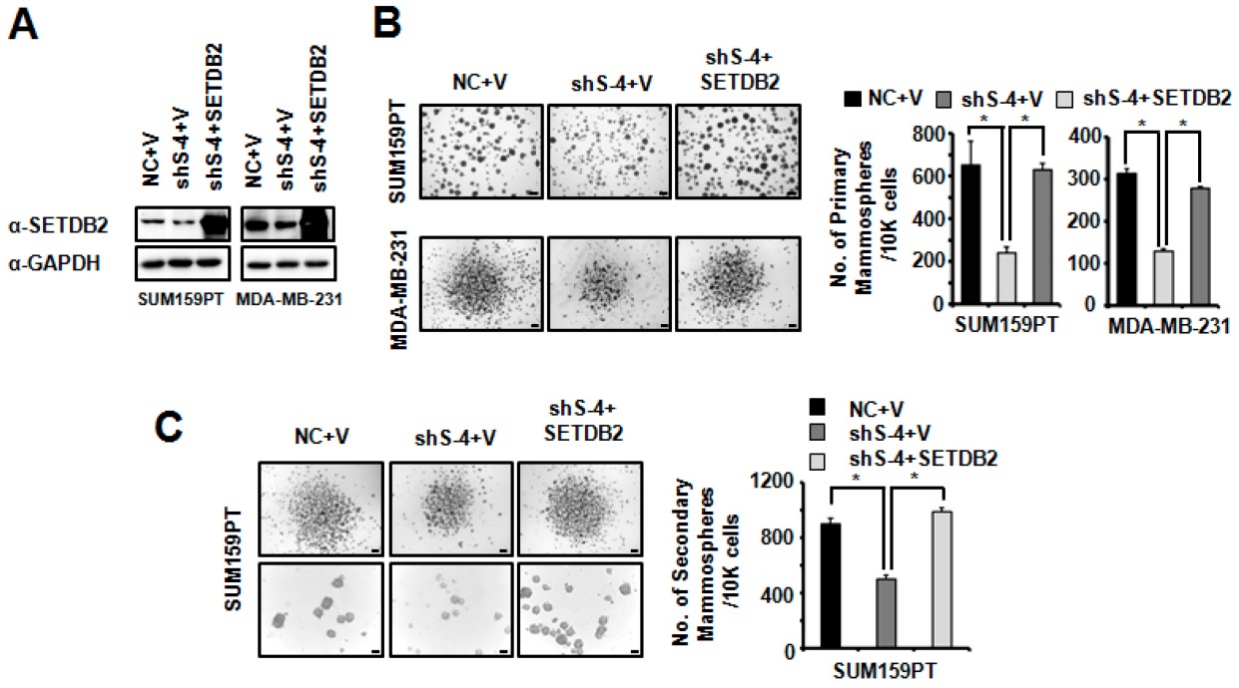
** Restored SETDB2 level rescued mammosphere formation.** (**A**) Western blot analysis of SETDB2 expression in NC, SETDB2-knockdown and rescued SUM159PT and MDA-MB-231 cells. (**B**) Representative microphotographs (bar, 100 μm and 200 μm) of primary mammospheres derived from NC, SETDB2-knockdown and SETDB2-rescued SUM159PT and MDA-MB-231 cells. Mammospheres with diameters greater than 50 μm were counted and quantified, as shown on the right. (**C**) Representative microphotographs (bar, 200 μm and 100 μm) of secondary mammospheres derived from NC, SETDB2-knockdown and SETDB2-rescued SUM159PT cells. Mammospheres with diameters greater than 50 μm were counted and quantified, as shown on the right. (data are presented as the mean ± SD of three independent experiments.*P < 0.05)

To further confirm the role of SETDB2 in BCSCs, we restored SETDB2 expression in SUM159PT and MDA-MB-231 SETDB2-knockdown cells. SETDB2 expression was examined by Western blot (**Figure 4A**). Then, we examined mammosphere formation in Control (NC+V), SETDB2-knockdown (sh S-4+V) and SETDB2-rescued (sh S-4+SETDB2) cells. After restoring SETDB2 expression, primary mammosphere formation was increased (**Figure 4B**), which was consistent with the SETDB2-knockdown data. In addition, the number and size of SUM159PT-derived secondary mammospheres were also rescued by SETDB2 restoration (**Figure 4C**). Thus, these data further indicate that SETDB2 is essential for BCSC maintenance.

### SETDB2 upregulates the Hedgehog pathway associated genes by interacting with and stabilizing ΔNp63α protein for breast cancer stem cell maintenance

**Figure 5:**
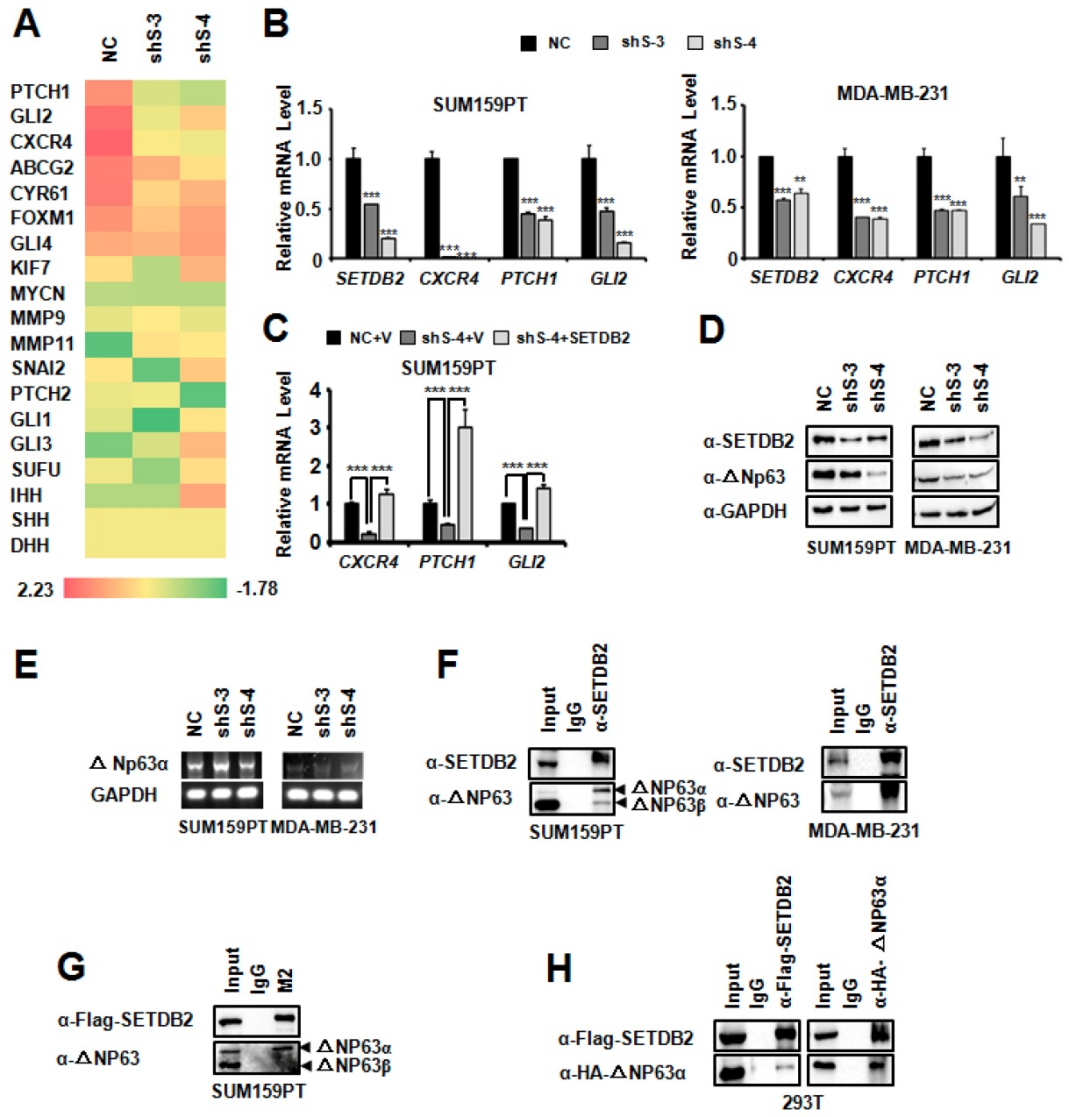
** SETDB2 interacted with ΔNp63α, and SETDB2 silencing downregulated Hedgehog pathway-associated genes and the ΔNp63α protein level.** (**A**) Heat map analysis of RNA-seq results in SETDB2-knockdown SUM159PT cells. (**B**) The mRNA levels of SETDB2 and Hedgehog pathway-associated genes (*CXCR4*, *PTCH1* and *GLI2*) were analyzed by Q-PCR in SETDB2-knockdown SUM159PT and MDA-MB-231 cells. (**C**) The mRNA levels of Hedgehog pathway-associated genes (*CXCR4*, *PTCH1* and *GLI2*) were analyzed by Q-PCR in NC, SETDB2-knockdown and SETDB2-rescued SUM159PT cells. (**D**) The protein levels of SETDB2 and ΔNp63α were analyzed by Western blot in SETDB2-knockdown SUM159PT and MDA-MB-231 cells. A GAPDH antibody was used as a loading control. (**E**) The level of ΔNp63α mRNA was analyzed by RT-PCR in SETDB2-knockdown SUM159PT and MDA-MB-231 cells. (**F**) Immunoprecipitation of SETDB2 showed that ΔNp63α endogenously interacted with SETDB2 in SUM159PT and MDA-MB-231 cells. (**G**) The interaction between exogenous Flag-SETDB2 and endogenous ΔNp63α were analyzed by anti-flag antibody immunoprecipitation in SETDB2-rescued SUM159PT cells. (**H**) SETDB2 and ΔNp63α were co-expressed in 293T cells. The exogenous interactions between SETDB2 and ΔNp63α were analyzed by co-immunoprecipitation and Western blotting. (NC: Negative Control; shS-3: shSETDB2-3; shS-4: shSETDB2-4; data are presented as the mean ± SD of three independent experiments. ***P < 0.005)

To understand the regulatory mechanism of SETDB2 in BCSCs, we performed an RNA-seq analysis. The heatmap showed that the expression of some Hedgehog signaling pathway-associated genes were decreased (**Figure 5A**). We further examined the transcription of the Hedgehog signaling pathway-associated genes *CXCR4*, *PTCH1* and *GLI2* 29-32 in SUM159PT and MDA-MB-231 SETDB2-knockdown cells by Q-PCR (**Figure 5B**). Compared with NC cells, the transcription levels of these genes were reduced in SETDB2-knockdown cells. However, the transcription inhibition of *CXCR4*, *PTCH1* and *GLI2* was rescued after SETDB2 expression was restored in SUM159PT cells (**Figure 5C**). These data indicate that SETDB2 upregulate the Hedgehog signaling pathway-associated genes.

As a histone H3K9 methyltransferase, SETDB2 can downregulate gene transcription due to its methyltransferase activity. However, in our systerm, SETDB2-knockdown downregulated the transcription of target genes, which suggests that SETDB2 can also upregulate gene transcription. We also examined the global histone H3K9 methylation level by Western blot in SUM159PT and MDA-MB-231 SETDB2-knockdown cells. SETDB2-knockdown did not change the global H3K9 methylation level (**Figure S3**). These data suggest that the transcription activity function of SETDB2 is not associated with its histone methyltransferase activity.

We noticed that* CXCR4*, *PTCH1* and *GLI2* are upregulated by ΔNp63α 12, 33. It was reported that SETDB1, which is also a member of the KMT1 sub-family 14, 34, can interact with ΔNp63α. SETDB1 silencing was found to downregulate the ΔNp63α protein level 35. We supposed that SETDB2 may also function via a similar mechanism, and thus we examined the ΔNp63α level by Western blot. Consistent with SETDB1, the ΔNp63α protein level was also decreased in SETDB2-knockdown SUM159PT and MDA-MB-231 cells (**Figure 5D**), while the transcription level of ΔNp63α did not decrease (**Figure 5E**). Immunoprecipitation showed that SETDB2 could endogenously interact with ΔNp63α in SUM159PT and MDA-MB-231 cells (**Figure 5F**). We also observed that SETDB2 weakly interacted with ΔNp63β in SUM159PT cells (**Figure 5F**). In addition, we examined the exogenous interaction between SETDB2 and ΔNp63α. In SETDB2-rescued SUM159PT cells, exogenous Flag-SETDB2 could co-immunoprecipitate endogenous ΔNp63α (**Figure 5G**). In 293T cells, co-expression of Flag-SETDB2 and HA-ΔNp63α also demonstrated that these two proteins can interact with each other (**Figure 5H**). These data suggested that SETDB2 interacts with △Np63α to stabilize ΔNp63α protein.

**Figure 6:**
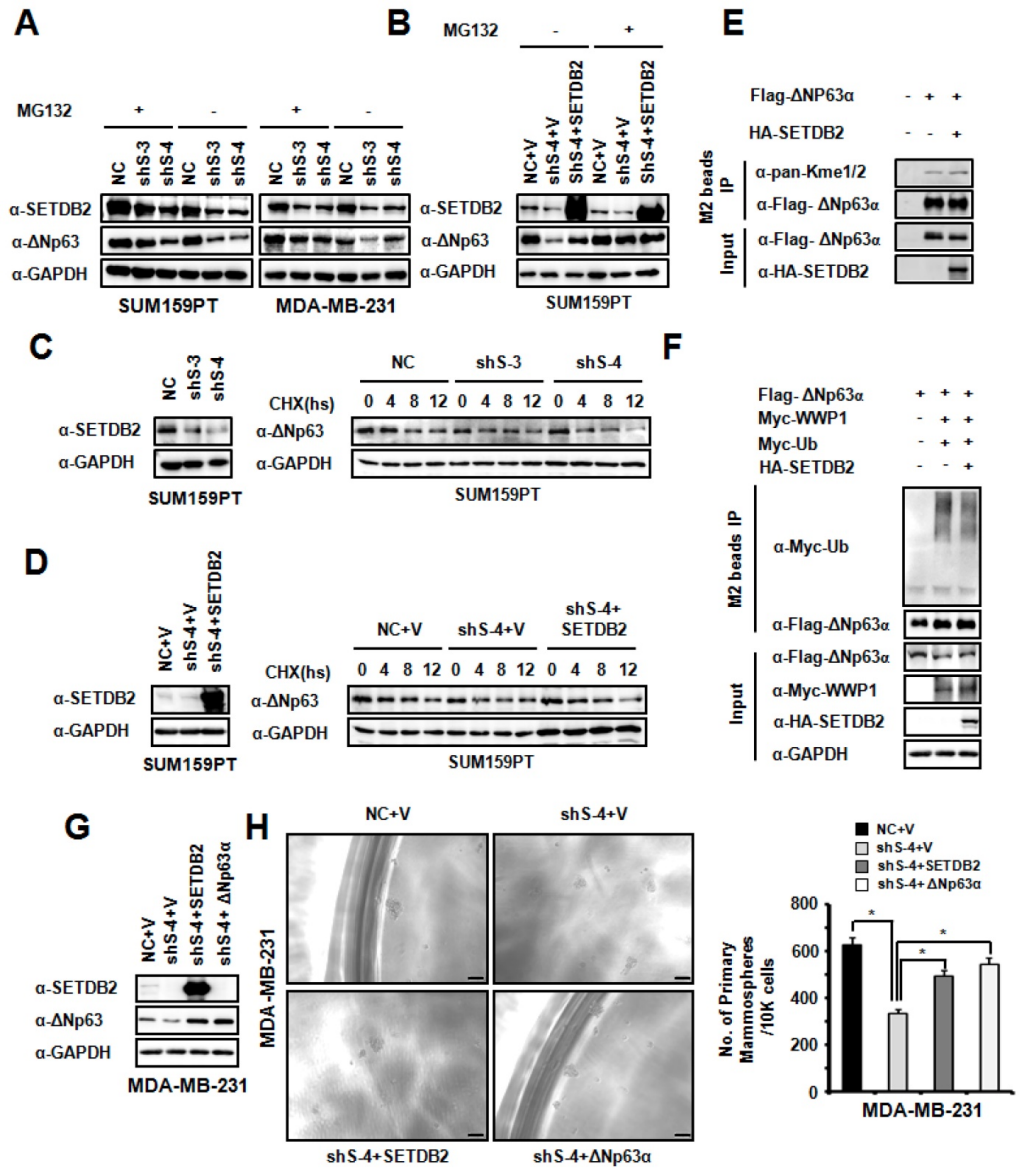
** SETDB2 protected ΔNp63α from proteasome-dependent degradation.** (**A**) Western blot analysis of SETDB2 and ΔNp63α in SETDB2-knockdown SUM159PT and MDA-MB-231 cells treated or untreated with MG132 for 12 hours. (**B**) Western blot analysis of SETDB2 and ΔNp63α in NC, SETDB2-knockdown and SETDB2-rescued SUM159PT cells treated or untreated with MG132 for 12 hours. (**C**) Western blot for SETDB2 in NC and SETDB2-knockdown SUM159PT cells. Western blot for ΔNp63α in NC and SETDB2-knockdown SUM159PT cells treated with CHX for 0, 4, 8, or 12 hours. (**D**) Western blot for SETDB2 in control (NC+V) SETDB2-knockdown (sh4+V) and SETDB2-rescued (sh4+SETDB2) SUM159PT cells. Western blot for ΔNp63α in control (NC+V) SETDB2-knockdown (sh4+V) and SETDB2-rescued (sh4+SETDB2) SUM159PT cells treated with CHX for 0, 4, 8, or 12 hours. (**E**) SETDB2 methylated ΔNp63α. 293T cell were co-transfected with expressing plasmids for Flag-ΔNp63α and HA-SETDB2. The IP was performed with the anti-FLAG M2 beads. Flag-ΔNp63α was detected by anti-Flag antibody. HA-SETDB2 was detected by anti-HA antibody. The methylation modified ΔNp63α proteins was detected by anti-pan-Kme1/2 antibody. (**F**) SETDB2 protect ΔNp63α from ubiquitination degradation. 293T cell were co-transfected with expressing plasmids for Myc-Ub, Myc-WWP1(E3 ubiquitin ligase of ΔNp63α), Flag-ΔNp63α and HA-SETDB2. The Immunoprecipitation was performed with the anti-FLAG M2 beads. The ubiquitin modified ΔNp63α proteins and WWP1 were detected by anti-Myc antibody. Flag-ΔNp63α was detected by anti-Flag antibody. HA-SETDB2 was detected by anti-HA antibody. Anti-GAPDH antibody used as loading control. (**G**) Western blot analysis of SETDB2 and ΔNp63α expression in control (NC+V), SETDB2-knockdown (sh4+V), SETDB2-rescued (sh4+SETDB2) and ΔNp63α-rescued (sh4+ΔNp63α) MDA-MB-231 cells. (**H**) Representative microphotographs (bar, 100 μm) of primary mammospheres derived from control (NC+V), SETDB2-knockdown (sh4+V), SETDB2-rescued (sh4+SETDB2) and ΔNp63α-rescued (sh4+ΔNp63α) MDA-MB-231 cells. Mammospheres with diameters greater than 50 μm were counted and quantified, as shown on the right. (data are presented as the mean ± SD of three independent experiments.*P < 0.05)

To explore the function of SETDB2 in ΔNp63α stabilization, we treated SETDB2-knockdown SUM159PT and MDA-MB-231 cells with the proteasome inhibitor MG132. The reduction in the ΔNp63α protein level mediated by SETDB2 silencing could be rescued by MG132 treatment (**Figure 6A**). We also treated SETDB2-knockdown and SETDB2-rescued SUM159PT cells with the proteasome inhibitor MG132. The reduction in the ΔNp63α protein level mediated by proteasome degradation could be rescued by restoration of the SETDB2 expression (**Figure 6B**). When we treated NC and SETDB2-knockdown SUM159PT cells with the protein synthesis inhibitor Cycloheximide (CHX), a significant reduction in the ΔNp63α protein half-life in SETDB2-knockdown cells was observed (**Figure 6C**). A significantly rescue in the ΔNp63α protein half-life was also observed in SETDB2-rescued SUM159PT cells with CHX treatment (**Figure 6D**). Some of SET family members can methylate non-histone protein and stabilized the substrate protein. So we co-expressed Flag-△Np63α and HA-SETDB2 in 293T cells, and performed immunoprecipitation with anti-FLAGs M2-agarose beads. The methylation level of ΔNp63α showed that SETDB2 increased the methylation level of ΔNp63α (**Figure 6E**). We also examinate the role of SETDB2 in ΔNp63α ubiquitination. We co-expressed Myc-Ub, Myc-WWP1(E3 ubiquitin ligase of ΔNp63α), Flag-ΔNp63α and HA-SETDB2 in 293T cells, and performed immunoprecipitation with anti-FLAGs M2-agarose beads under a denaturing condition. The ubiquitination level of ΔNp63α showed that SETDB2 reduced the ubiquitination level of ΔNp63α which induced by E3 ligase WWP1(**Figure 6F**).

To further confirm whether SETDB2 promoted breast cancer stem cell maintenance by ΔNp63α, we restored ΔNp63α expression in MDA-MB-231 SETDB2-knockdown cells. ΔNp63α and SETDB2 expression was examined by Western blot (**Figure 6G**). Then, we examined mammosphere formation in Control (NC+V), SETDB2-knockdown (sh S-4+V), SETDB2-rescued (sh S-4+SETDB2) and ΔNp63α-rescued (sh S-4+△Np63α) cells. After restoring ΔNp63α expression, the mammospheres formation was increased, which was consistent with the SETDB2-rescued group (**Figure 6H**).

Thus, all these data indicated that SETDB2 interacts with ΔNp63α, methylates and stabilizes ΔNp63α protein for breast cancer stem cell maintenance.

**Figure 7:**
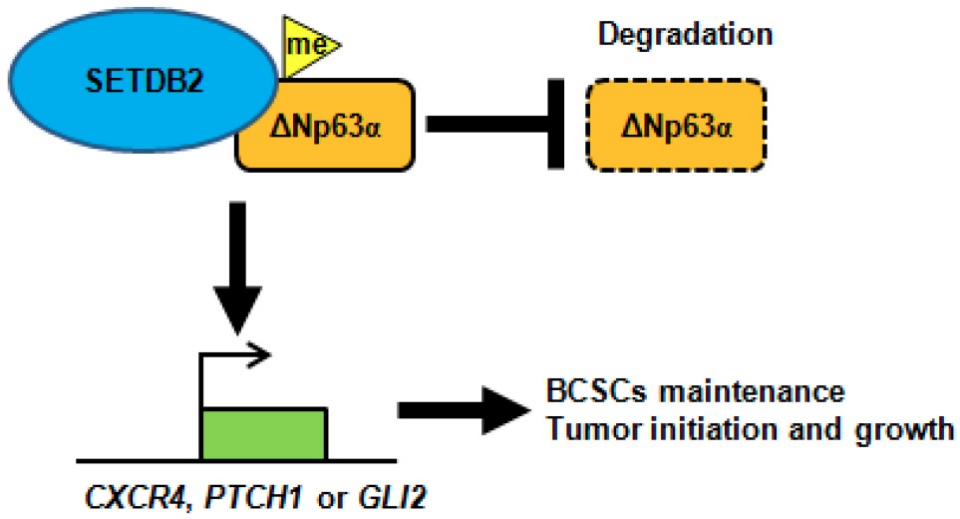
** A schematic of how SETDB2 promoted breast cancer stem cell maintenance, tumor initiation and growth.** SETDB2 interacts with ΔNp63α, methylates and stabilizes ΔNp63α, and upregulates the Hedgehog pathway-associated genes *CXCR4*, *PTCH1* and *GLI2* to promote stem cell maintenance, tumor initiation and growth.

In conclusion, SETDB2 interacts with ΔNp63α, methylates and stabilizes ΔNp63α to upregulate the transcription of the Hedgehog signaling pathway-associated genes* CXCR4*, *PTCH1* and *GLI2*, which promote breast cancer stem cell maintenance and tumor initiation (**Figure 7**).

## Discussion

In our study, we discovered a novel function of SETDB2 in breast cancer stem cell maintenance. First, we found that the SETDB2 expression level was significantly increased in mammospheres derived from breast cancer cell lines. Silencing SETDB2 significantly decreased the percentage of ALDH^+^ cells and mammosphere formation *in vitro*. Silencing SETDB2 also decreased breast tumor initiation and growth *in vivo*. Restoration of the SETDB2 expression level rescued mammosphere formation in breast cancer cells. The mechanistic analysis suggested that SETDB2 upregulated the transcription of the ΔNp63α downstream Hedgehog pathway-associated genes-- *CXCR4*, *PTCH1* and *GLI2*. SETDB2 also interacted with ΔNp63α, methylated and stabilized ΔNp63α protein. Restoring ΔNp63α expression rescued the breast cancer stem cell maintenance defect mediated by SETDB2-knockdown. Thus, our study reveals an essential function of SETDB2 in breast cancer stem cell maintenance.

SETDB2 is a histone methyltransferase grouped in the KMT1 sub-family. In the KMT1 sub-family, SUV39H1 and G9a interact with Snail and repress E-cadherin transcription by their histone H3K9 methyltransferase activity to regulate epithelial-mesenchymal transition in breast cancer cells 36, 37. SETDB1 is recruited to the Snail promoter by Smad3, which regulates Snail1 expression and epithelial-mesenchymal transition by its histone H3K9 methyltransferase activity 38, 39. All these reports revealed the transcription repression function of the KMT1 sub-family members. This transcription repression function is in turn associated with histone H3K9 methyltransferase activity. However, Some of SET family members can methylate non-histone protein and stabilized the substrate protein to active gene transcription. SET7/9 can methylate p53 40 and LIN28A 41, and increase their nuclear retention and protein stability. SET7/9 can also methylate and stabilize ERα 42 and Gli3 43, and increase their stability and DNA binding ability, resulting in target genes transactivation. In our study, we found that SETDB2 can methylate and stabilize ΔNp63α, and active ΔNp63α target gene transcription. These results suggest SETDB2 also has transcription activity function through its non-histone methylation.

p63 is a member of the p53 protein family. Like p53, ubiquitination is also a common pathway for the negative regulation of p63. The stability of ΔNp63α, which is the major isoform of p63, is very important for its function. The primary pathway of ΔNp63α degradation is mediated by a proteasome-dependent pathway. In our study, we also found that SETDB2 can interact with ΔNp63α, methylate and stabilize ΔNp63α. We have found that SETDB2 can inhibit the ubiquitination of ΔNp63α mediated by WWP1. While how does SETDB2 inhibit the ubiquitination and regulate the stability of ΔNp63α need to discover: (i) In proteasome-dependent pathway, besides WWP1, there are several other E3 ligases such as Nedd4 44, Itch 45 and Fbw7 46 also essential for this process. SETDB2 may also inhibit the ubiquitination mediated by other E3 ligases. (ii) In addition, phosphorylation which mediated by several kinases such as ATM 47, CDK2 47 and p38 48, also play key roles in ΔNp63α protein degradation. Moreover, other proteins that function as regulators or cofactors of E3 ligases and kinases can also regulate ΔNp63α stabilization 44, 46, 48, 49. The interaction site between ΔNp63α and E3 ubiquitin ligases, kinases and associated factor may be blocked by SETDB2 binding. (iii) It was reported that histone methyltransferases can also methylate non-histone proteins. SET7 can methylate p53 40, ERα 42, LIN28A 41 and Gli3 43 to promote protein stability. SUV39H2, which is also a homolog of SETDB2, can methylate LSD1 to enhance its stability 50. We found SETDB2 can methylate ΔNp63α, which suggested that the ΔNp63α methylation may crosstalk with phosphorylation and ubiquitination to protect ΔNp63α from degradation. (iv) Protein ubiquitination catalyzed by E3 ligases can be reversed by deubiquitinating enzymes (DUBs) to prevent protein from degradation. Some core stem cell transcription factors, such as Oct3/4, c-Myc, Sox2, Klf4, Nanog, and LIN28 can be ubiquitinate and deubiquitinate in stem cell maintenance and differentiation 51. ΔNp63α as a key transcription factor in breast cancer stem cell may also be deubiquitinated by DUBs. In our study, SETDB2 interacts with ΔNp63α. SETDB2 may recruit the DUBs to protect ΔNp63α from degradation.

Taken together, SETDB2 interacts with ΔNp63α, methylates and stabilizes the ΔNp63α protein to upregulate the Hedgehog pathway-associated genes* CXCR4*, *PTCH1* and *GLI2*, which promote stem cell maintenance, tumor initiation and growth (**Figure 7**). Our study reveals a novel function of SETDB2 in breast cancer stem cells.

## Supplementary Material

Supplementary figures and tables.

## Abbreviations

BCSCs: Breast cancer stem cells
ALDH: Aldehyde dehydrogenase
CCK-8: Cell Counting Kit-8
Q-PCR: Quantitative Polymerase Chain Reaction
RT-PCR: Reverse Transcription Polymerase Chain Reaction
SD: Standard deviation
SEM: Standard Error of the Mean
NC: Negative control
TSSs: transcription start sites
TAD: transactivation domain
SHH: Sonic Hedgehog
GLI2: GLI family zinc finger 2
PTCH1: Patched1
CHX: Cycloheximide
ELDA: extreme limiting dilution analysis
