# Supplementary material for: SETDB2 promoted breast cancer stem cell maintenance by interaction with and stabilization of ΔNp63α protein
Source: Int J Biol Sci. 2020 May 18;16(12):2180–91. doi: 10.7150/ijbs.43611 (PMC7294945; doi:10.7150/ijbs.43611)
Supplement: Supplementary file 1 — Supplementary figures and tables. [file ijbsv16p2180s1.pdf]

A

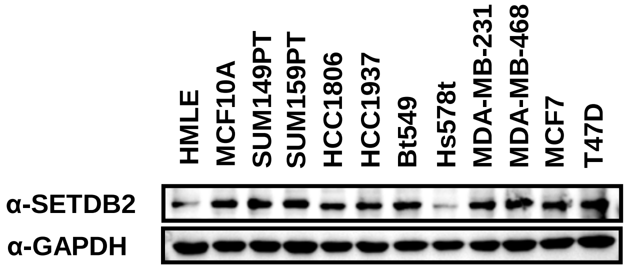
HMLE
MCF10A
SUM149PT
SUM159PT
HCC1806
HCC1937
Bt549
Hs578t
MDA-MB-231
MDA-MB-468
MCF7
T47D
α-SETDB2
α-GAPDH

Figure S1. SETDB2 expression level in breast cancer cell lines. (**A**) Western blot was used to analyze the SETDB2 expression level in breast cancer cell lines.

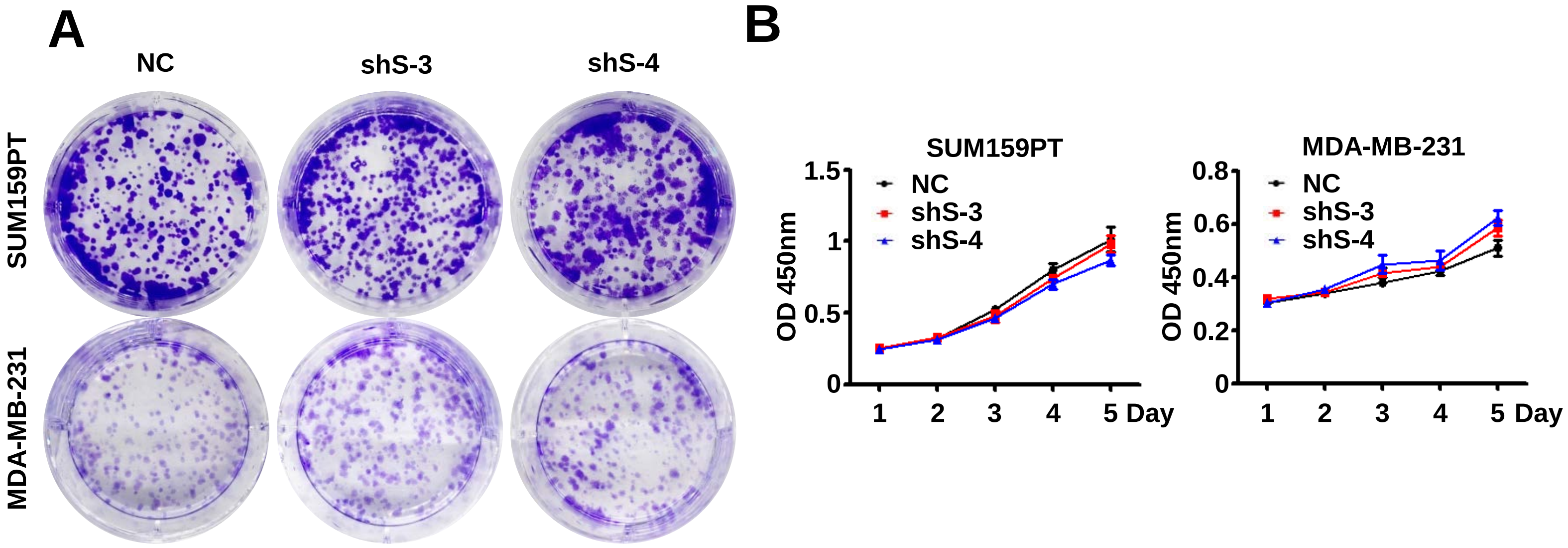
A
NC
shS-3
shS-4
SUM159PT
MDA-MB-231
B
SUM159PT
MDA-MB-231
OD 450nm
NC
shS-3
shS-4
1.5
1
0.5
0
0.8
0.6
0.4
0.2
1
2
3
4
5 Day

Figure S2. SETDB2 has negligible effect on breast cancer cell proliferation. (**A**) The colony formation assay in SETDB2-knockdown SUM159PT and MDA-MB-231 cell lines. (**B**) The CCK-8 assay in SETDB2-knockdown SUM159PT and MDA-MB-231 cell lines.

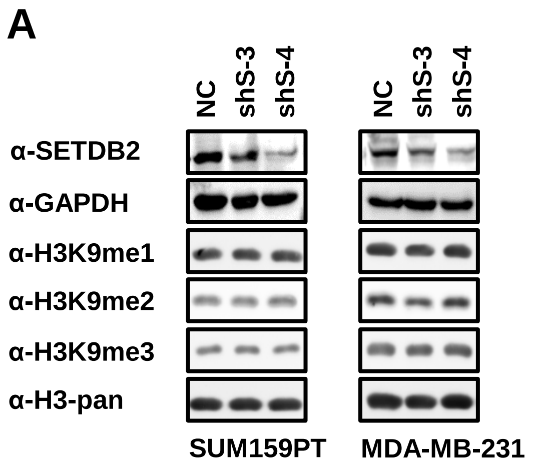
A
NC
shS-3
shS-4
NC
shS-3
shS-4
α-SETDB2
α-GAPDH
α-H3K9me1
α-H3K9me2
α-H3K9me3
α-H3-pan
SUM159PT
MDA-MB-231

Figure S3. SETDB2 has negligible effect on the global histone H3K9 methylation level in breast cancer cells. (**A**) Western blot was used to determine the SETDB2, H3, H3K9me1, H3K9me2 and H3K9me3 levels in SETDB2-knockdown SUM159PT and MDA-MB-231 cell lines. An anti-H3-pan and anti-GAPDH antibody was used as the loading control. The anti-SETDB2 antibody showed the knockdown efficiency in SUM159PT and MDA-MB-231 cell lines.

Table S1: SETDB2 sequence.

GGTACCATGGGAGAGAAGAACGGCGACGCCAAGACCTTTTGGATGGAGCT<br>CGAGGACGACGGCAAGGTGGACTTCATCTTCGAGCAGGTGCAGAACGTGC<br>TGCAGAGCCTGAAGCAGAAGATCAAGGACGGCAGCGCCACCAACAAGGA<br>GTACATCCAGGCCATGATCCTGGTGAACGAGGCCACAATCATCAACAGCAG<br>CACCAGCATCAAGGGCGCCTCTCAGAAGGAAGTGAACGCCCAGAGCAGC<br>GACCCTATGCCAGTGACCCAGAAGGAGCAGGAGAACAAGAGCAACGCCTT<br>CCCCAGCACCTCTTGCGAGAACAGCTTCCCCGAGGACTGCACCTTCCTGA<br>CCACCGAGAACAAGGAGATCCTGAGCCTGGAGGACAAGGTGGTGGACTTC<br>CGGGAGAAGGACAGCAGCAGCAACCTGAGCTACCAGAGCCACGATTGTAG<br>CGGCGCTTGCCTGATGAAGATGCCCCTGAACCTGAAGGGAGAGAACCCTC<br>TGCAGCTGCCCATCAAGTGCCACTTCCAGAGGAGGCACGCCAAGACAAAC<br>TCTCACAGCAGCGCCCTGCACGTGTCTTACAAGACCCCTTGCGGCAGGAG<br>CCTGAGAAACGTGGAGGAGGTGTTCCGCTACCTGCTGGAGACCGAGTGCA<br>ACTTCCTGTTCACCGACAACTTCAGCTTCAACACCTACGTGCAGCTGGCCA<br>GGAACTACCCCAAGCAGAAGGAGGTCGTGTCCGACGTGGACATCAGCAAC<br>GGAGTGGAGAGCGTGCCCATCAGCTTCTGCAACGAGATCGACAGCCGGAA<br>GCTGCCCCAGTTCAAGTACCGGAAAACCGTCTGGCCCAGAGCCTACAACC<br>TGACCAACTTCAGCAGCATGTTCACCGACTCTTGCGACTGCTCCGAGGGGT<br>GCATTGACATCACCAAGTGCGCTTGCCTGCAGCTGACCGCTAGAAACGCCA<br>AGACCAGCCCTCTGAGCAGCGACAAGATCACCACCGGCTACAAGTACAAG<br>CGGCTGCAGAGGCAGATCCCCACAGGCATCTACGAGTGCAGCCTCCTGTG<br>CAAATGCAACCGCCAGCTCTGCCAGAACAGAGTGGTGCAGCACGGACCTC<br>AGGTCCGACTGCAGGTGTTCAAGACCGAGCAGAAGGGTTGGGGCGTGAG<br>ATGTCTGGACGACATCGACAGGGGCACCTTCGTCTGCATCTACTCCGGCAG<br>GCTGCTGTCTCGGGCCAACACCGAGAAGAGCTACGGAATCGACGAGAACG<br>GCAGGGACGAGAACACCATGAAGAACATCTTCAGCAAGAAGCGGAAGCT<br>GGAGGTGGCTTGCAGCGATTGCGAGGTGGAAGTGCTGCCACTGGGACTGG<br>AAACCCACCCTAGAACCGCCAAGACCGAGAAGTGCCCCCCCAAGTTCAGC<br>AACAACCCCAAGGAGCTGACCGTGGAGACCAAGTACGACAACATCAGCCG<br>GATCCAGTACCACAGCGTGATCAGGGACCCAGAGAGCAAGACCGCCATCT<br>TCCAGCACAACGGCAAGAAGATGGAGTTCGTGTCCAGCGAGAGCGTGACA<br>CCAGAGGACAACGACGGCTTCAAGCCTCCTAGGGAGCACCTGAACAGCAA<br>GACCAAGGGAGCCCAGAAGGACAGCAGCAGCAACCACGTGGACGAGTTC<br>GAGGACAACCTGCTGATCGAGAGCGACGTGATCGACATCACCAAGTACCG<br>GGAGGAGACCCCTCCTAGAAGTCGCTGTAACCAGGCCACCACCCTGGACA<br>ACCAGAACATCAAGAAGGCCATCGAGGTGCAGATCCAGAAGCCCCAGGAG<br>GGAAGAAGCACAGCTTGCCAGAGGCAGCAGGTCTTCTGCGACGAAGAGC<br>TGCTGAGCGAGACCAAGAACACCAGCAGCGACAGCCTGACCAAGTTCAA<br>CAAGGGCAACGTGTTCCTGCTGGACGCCACAAAGGAGGGAAACGTGGGC<br>CGGTTCCTGAACCACTCTTGTTGCCCCAACCTGCTGGTGCAGAACGTGTTC<br>GTGGAGACCCACAACCGGAACTTCCCTCTGGTGGCCTTCTTCACCAACCG<br>CTACGTGAAGGCCAGAACCGAGCTGACTTGGGACTACGGCTACGAAGCCG<br>GAACAGTGCCCGAGAAGGAGATCTTTTGCCAGTGCGGGGTCAACAAGTGC

CGGAAGAAGATCCTGGACTACAAGGACGACGACGACAAGTAGGAATTC

Table S2: Q-PCR and RT-PCR primer sequences.

Primer Name	Sequences (5’-3’)
SETDB2-S:	CTGCCAATCAAATGTCACTTCC
SETDB2-AS:	CCACGTTTCGTAGACTCCTTCC
CXCR4-S:	TCTTCCTGCCCACCATCTACTC
CXCR4-AS:	GTACTTGTCCGTCATGCTTCTCAG
PTCH1-S:	TCAACTGGAACGAGGACAAAGC
PTCH1-AS:	CGTGGTGGTGAAGGAAAGCA
GLI2-S:	TCTGGGGTCAGCCTTTGGA
GLI2-AS:	CTGCTTGTTCTGGTTGGTGTCA
▵Np63α-RT-S:	GAAAACAATGCCCAGACTCAAT
▵Np63α-RT-AS:	AGCCCAACCTCGCTAAGAAA
